# Glycine receptor mutants of the mouse: what are possible routes of inhibitory compensation?

**DOI:** 10.3389/fnmol.2012.00098

**Published:** 2012-10-31

**Authors:** Natascha Schaefer, Nicolas Vogel, Carmen Villmann

**Affiliations:** ^1^Emil Fischer Center, Institute of Biochemistry, University Erlangen-NuernbergErlangen, Germany; ^2^Institute for Clinical Neurobiology, University of WuerzburgWuerzburg, Germany

**Keywords:** GlyRs, synaptic inhibition, spontaneous mouse mutants, knockout mice, hyperekplexia, rescue

## Abstract

Defects in glycinergic inhibition result in a complex neuromotor disorder in humans known as hyperekplexia (OMIM 149400) with similar phenotypes in rodents characterized by an exaggerated startle reflex and hypertonia. Analogous to genetic defects in humans single point mutations, microdeletions, or insertions in the *Glra1* gene but also in the *Glrb* gene underlie the pathology in mice. The mutations either localized in the α (*spasmodic, oscillator, cincinnati, Nmf11*) or the β (*spastic*) subunit of the glycine receptor (GlyR) are much less tolerated in mice than in humans, leaving the question for the existence of different regulatory elements of the pathomechanisms in humans and rodents. In addition to the spontaneous mutations, new insights into understanding of the regulatory pathways in hyperekplexia or glycine encephalopathy arose from the constantly increasing number of knock-out as well as knock-in mutants of GlyRs. Over the last five years, various efforts using *in vivo* whole cell recordings provided a detailed analysis of the kinetic parameters underlying glycinergic dysfunction. Presynaptic compensation as well as postsynaptic compensatory mechanisms in these mice by other GlyR subunits or GABA_A_ receptors, and the role of extra-synaptic GlyRs is still a matter of debate. A recent study on the mouse mutant *oscillator* displayed a novel aspect for compensation of functionality by complementation of receptor domains that fold independently. This review focuses on defects in glycinergic neurotransmission in mice discussed with the background of human *hyperekplexia en route* to strategies of compensation.

## Introduction

Synaptic inhibition is mediated by activation of glycine receptors (GlyRs) and γ-aminobutyric acid (GABA) receptors. GlyRs mediate fast inhibitory neurotransmission in the adult spinal cord, which is important for motor control. Functional impairments of spinal GlyRs or associated proteins lead to the rare neuromotor disorder hyperekplexia (Startle disease, Stiff Baby Syndrome, OMIM #149400) in humans with similar phenotypes in rodents. In hyperekplexia, affected neonates exhibit exaggerated startle responses and muscle stiffness following an unexpected acoustic noise or a tactile stimulus, which may result in fatal apnea. During early childhood the muscle tone returns to normal levels, but startling persists into the adulthood (Andermann et al., 1980; Shiang et al., 1993; Becker et al., 2008). Predominantly, single point mutations in the *GLRA1* gene encoding the GlyR subunit α1 (GlyRα1) underlie the pathology in humans. Analogous genetic defects have been observed in mice. The mutations are either localized in the α (*spasmodic, oscillator, cincinnati, Nmf11*) or the β (*spastic*) subunit of the GlyR. Recently, other animal models of glycinergic dysfunction have been reported in zebrafish (*bandoneon, shocked*) and cow (*CMD2*). Among the mouse mutations, *spasmodic*, *spastic, oscillator*, and *cincinnati* harbor spontaneous mutations, the *Nmf11* mouse mutant instead results from chemical induction (Buckwalter et al., 1994; Kingsmore et al., 1994; Mulhardt et al., 1994; Ryan et al., 1994; Holland et al., 2006; Traka et al., 2006). These mouse models of GlyR dysfunction have served as excellent systems for studying the pathomechanisms of the corresponding human neurological disease. It turned out, however, that the phenotype in mice is much more severe than in humans, e.g., a functional null allele is lethal in mice (*oscillator*) but not in humans, arguing for different compensatory mechanisms in both organisms (Tsai et al., 2004; Becker et al., 2006). The response to clonazepam treatment is another example for differences in compensatory effects. In patients, startle attacks are reduced and a normal muscle tone could be reached following clonazepam treatment. The benzodiazepine clonazepam enhances GABA-gated chloride currents upon binding to GABA_A_ receptors exhibiting an up-regulation of GABAergic neurotransmission as one possible compensatory mechanism at least in humans. In contrast, no similar mechanism seems to exist in mice after clonazepam treatment. Here, background effects as well as the existence of other so far unidentified regulatory proteins or modulators of the disease are under discussion (Buckwalter et al., 1994; Becker et al., 2006; Molon et al., 2006). In the two mouse models *spastic* and *oscillator* a functional rescue has been shown using different approaches *in vitro* and *in vivo*. With a transgenic approach in the mouse mutant *spastic* the phenotype was rescued by restoring the β expression levels toward sufficient amounts for proper functioning of glycinergic synapses (Hartenstein et al., 1996). Using a complementation strategy, α1 levels came up in spinal cord neurons isolated from homozygous *oscillator* mice (Villmann et al., 2009a).

This review will discuss the various mouse models of glycinergic disinhibition to understand the pathomechanisms and regulatory pathways of the neuromotor disorder hyperekplexia. With the help of knock-out, and knock-in studies of GlyR subunits, including novel strategies such as rescue experiments new perspectives of pre- or postsynaptic compensation will be elaborated.

## Glycinergic synapses and neuromotor behavior

Glycinergic neurons appear during development in the vertebrate central nervous system (CNS) from embryonic day 12.5 (E12.5) on in the somata of ventral horn cells in the spinal cord, followed by cells in the dorsal horn one day later (Allain et al., 2006; Chalphin and Saha, 2010). Prominent GlyR immunoreactivity has been also detected in the ventral and dorsal horns of the spinal cord, the spinal trigeminal nuclear complex, the dorsal motor nucleus of the vagus nerve, and the hypoglossal nucleus (Rampon et al., 1996; Baer et al., 2009). Further GlyR expression in the adult CNS has been determined in midbrain, caudal and rostral pons, rostral medulla oblongata, retina, different nuclei of the forebrain, and the cochlea (Greferath et al., 1994; Baer et al., 2003; Waldvogel et al., 2007; Dlugaiczyk et al., 2008).

In early stages of development GABAergic transmission predominates over glycinergic transmission. At this time both neurotransmitters depolarize neurons (Gonzalez-Forero and Alvarez, 2005; Scain et al., 2010). In the neonatal brain many inhibitory synapses initially are mixed GABAergic and glycinergic, which changes during CNS maturation between P5 and P14 depending on the CNS area (Jonas et al., 1998; Kotak et al., 1998). The switch toward glycinergic transmission has been observed in many spinal and brainstem synapses, e.g., in the lumbar spinal cord and in the lateral superior olive of young rodents (Gao et al., 2001; Nabekura et al., 2004; Muller et al., 2006; Scain et al., 2010). But there are also regions in which GABAergic neurotransmission dominates in the developing brain for example in the collicular neurons (Meier et al., 2002).

**Figure 1:**
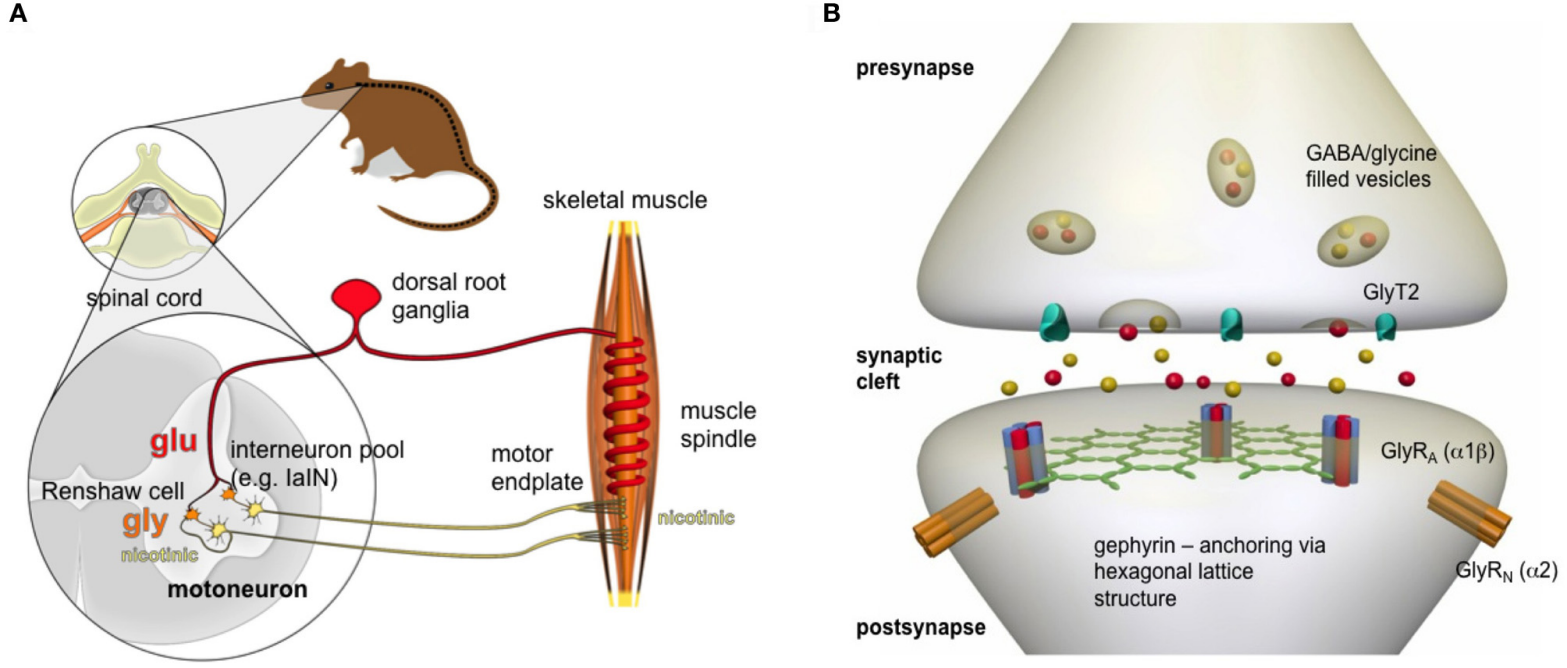
**Nerve-muscle circuit**. **(A)** Following an acoustic signal or a tactile stimulus, sensory neurons (red), which are located in dorsal root ganglia, release glutamate that binds to appropriate receptors localized at the motoneuronal membrane (yellow), thereby leading to membrane depolarization. The motoneuron fires action potentials toward the neuromotor endplate where the signal is transmitted again via an excitatory (nicotinic) neurotransmitter acetylcholine. The efficiency of the system is enabled by the action of glycine receptors. Inhibitory interneurons (orange, IaIN, or Renshaw cells) in the spinal cord get activated by either acetylcholine released from collateral axons of the motoneuron or glutamate released from primary afferents, form inhibitory synapses with the membrane of motoneurons. Renshaw cells release glycine that binds to the GlyRs localized at the motoneuronal membrane leading to hyperpolarization of the membrane. Thus, functional GlyRs lead via the feedback control loop to only that amount of acetylcholine release, which is sufficient to reach the threshold for an action potential that propagates along the muscle fiber. **(B)** GlyRs are localized at the postsynaptic membrane in a pentameric receptor configuration of 2α and 3β subunits. The intracellular scaffold protein gephyrin clusters the GlyRs at the membrane thereby forming a hexagonal lattice structure. The activation of voltage-gated calcium channels present in the presynapse lead to calcium influx. As a consequence, mixed GABA/glycine vesicles fuse to the presynaptic membrane and release their content into the synaptic cleft. Following binding of glycine to the GlyR, a conformational change leads to ion channel opening and chloride ion influx.

Important in the maturation of ventral inhibitory synapses are the developing interneurons (e.g., IaIN, V2b, Renshaw cells), which possess diverse roles in shaping locomotor patterns. These interneurons are assigned to enable reciprocal (Ia inhibitory neurons), feedback (Renshaw cells), and feed-forward (presynaptic sensory) inhibition (Figure 1) (Siembab et al., 2010). Compared to other ventral interneurons, Renshaw cells become greatly potentiated inhibitory synaptic inputs during postnatal development as a consequence of functional adaptation. Renshaw cell-mediated recurrent inhibition allows the control of the dynamic behavior of motoneuron (MN) firing (Gonzalez-Forero and Alvarez, 2005; Alvarez and Fyffe, 2007). Neuromotor behavior is fine-tuned by recurrent inhibition, which involves the action of GlyRs binding the inhibitory neurotransmitter glycine. The activation of GlyRs leads to chloride influx and therefore hyperpolarization of the motoneuronal membrane restoring the balance between excitation and inhibition (Figure 1). This feedback circuit emphasizes the importance of glycinergic inhibition in the adult spinal cord and brainstem.

Disturbances in GlyR inhibition due to a single amino acid substitution in one of the GlyR subunits give rise to a different MN-firing pattern associated with overactivation of the muscle and muscle stiffness. Such symptoms are observed in patients suffering from hyperekplexia. These patients carry a mutant allele with either a single nucleotide polymorphism (SNP) leading to an amino acid exchange at the protein level, deletions of various exons or nonsense mutations (Harvey et al., 2008). Dominant mutations have been shown to result in differences in physiological properties of the ion channel itself. The majority is localized within the ion channel domain itself and adjacent loop structures (Lynch et al., 1997). Recessive mutations showed up with a disturbed biogenesis with regard to trafficking and accumulation in the endoplasmic reticulum. In addition, the stability of these mutants is highly affected (Villmann et al., 2009b). In homozygous mutant mice point mutations within the *Glra1* and the *Glrb* gene result in similar phenotypes with typical startle attacks, massive tremor and rigidity from postnatal day 15 on. This has been shown for spontaneous mouse mutants as well as for knock-in animals carrying point mutations that were identified in human patients (Findlay et al., 2003; Borghese et al., 2012). Between postnatal days 15 and 21 the disease is highly progressive and lethal for the majority of mutants. The lethal phenotype has been noted for mutations in the α1 and β subunit, both of which are part of the adult receptor complex. In contrast, α2 or α3 knockout animals do show a rather mild phenotype (Harvey et al., 2004; Young-Pearse et al., 2006). Thus, compensatory mechanisms get rather initialized by a complete loss of protein function than following the expression of misfolded receptor proteins accumulating in the cell, respectively.

## Glycine receptors—ligand-gated chloride channels

GlyRs are members of the superfamily of Cys-loop receptors (CLRs) as the nicotinic acetylcholine receptors, the 5HT_3_ receptor, and GABA_A/C_ receptors (Lynch, 2009). The overall topology of a single subunit with a conserved disulphide bridge in the N-terminus is common to all members of the CLR family (Lynch, 2004). Characteristic features are a short C-terminus and a highly structured N-terminus, which exhibits a high homology to the acetylcholine binding protein of the pond snail *Lymnea stagnalis*, whose structure was solved by X-ray crystallography with a resolution of 2.7 Å (Brejc et al., 2001). The N-terminal domain displays an immunoglobuline-like structure characterized by a short α-helical domain in the far N-terminus followed by 10 β-sheets. Meanwhile, the X-ray structure of other members of the CLR superfamily has been solved, e.g., GLIC and ELIC (Hilf and Dutzler, 2008, 2009; Bocquet et al., 2009), two members of prokaryotic origin, and the GluCl, a glutamate-gated chloride channel from *C. elegans* with 34% protein identity to the GlyR structure (Hibbs and Gouaux, 2011). Furthermore, each subunit comprises 4 transmembrane domains (TMs) connected by intra- or extracellular loop structures. TM2 forms the ion channel pore. The highest diversity among GlyR subunits and also among different families of CLRs lies in the large TM3-4 loop. This loop is thought to be important for biogenesis and posttranslational modifications (Sadtler et al., 2003; Breitinger et al., 2009; Villmann et al., 2009a).

Upon agonist binding toward the ligand-binding cavity (Brejc et al., 2001; Unwin, 2005) an intrinsic chloride channel opens. The structural data have shown that amino acids from N-terminal loop structures, loops A, B, and C from one subunit (plus side or principal face) together with loops D, E, and F from another subunit (minus side or complementary face) form the ligand binding cavity between different subunits. The adult receptor channel complex consists of 5 subunits assembling into a rosette-like heteropentamer of two α and three β subunits both of which are involved in ligand binding (Grudzinska et al., 2005; Dutertre et al., 2012). The opening of the GlyR ion channel pore follows a ligand-induced (starting with the highest affinity: glycine > β-alanine > taurine) transition process (Lynch, 2004).

## GlyR subunit diversity and developmental regulation

At present four GlyRα-subunits encoded by different genes are known, as well as one single β-subunit. Alternative splicing of the α subunits as well as the β-subunit results in further diversity of GlyR complexes (Malosio et al., 1991a; Kuhse et al., 1993; Nikolic et al., 1998; Harvey et al., 2004; Heinze et al., 2007; Oertel et al., 2007).

GlyRα1 exists in two splice variants (α1ins described for rat, mice, and humans) with one harboring additional 8 amino acids in the TM3-4 loop (Malosio et al., 1991a; Strausberg et al., 2002). Both α1 variants do not differ in physiological characteristics in transfected cell lines. The α1 subunit mRNA in rats is first detectable at postnatal day 5 and increases constantly into the adulthood (Malosio et al., 1991b; Sato et al., 1991). The expression of the α1 is most prominent in brain nuclei and spinal cord; some immunoreactivity was observed in the superior and inferior folliculi and regions of the hypothalamus and in the thalamus (Sato et al., 1991). The mRNA of α2 has been detected first around embryonic day E15 and decreased after birth (Malosio et al., 1991b). Originally, a developmental switch from a neonatal isoform GlyR_N_ composed of α2 homomers to the adult isoform (GlyR_A_) α1β heteromers was postulated. This switch was proposed to be complete by postnatal day 20. For GlyRα2 also two alternative splice variants with alternative exons 3 have been described, either exon 3A or 3B. The resulting proteins differ in only two amino acid residues in the extracellular N-terminal part of the receptor (Kuhse et al., 1991). Recent findings suggest that the α2 expression persists into adulthood in retina, cerebellum, and auditory brain stem (Piechotta et al., 2001; Young and Cepko, 2004; Heinze et al., 2007; Nobles et al., 2012). GlyR3α also exists in two splice variants, differing in a 15 amino acid insertion in the TM3-4 loop (α3L and α3K). Both variants show similar distribution which is comparable to the α1 subunit, but with significantly less intensity. In contrast to the α1 splice isoforms α3L and α 3K differ in ion channel properties, e.g., in desensitization behavior with α3K forming fast desensitizing channels and α3L representing the slower desensitizing receptor complexes (Nikolic et al., 1998). In addition, RNA editing has been shown to increase the diversity of GlyRα3 (Meier et al., 2005). The α3 variant P185L emerges from cytidine 554 deamination (C554U). Neurons harboring α3P185L assign high glycine potency and non-desensitized receptors, which are prerequisites for tonic inhibition (Stell and Mody, 2002). The α4 subunit has been found in the adult mouse retina where it clusters together with synaptic markers such as bassoon or gephyrin (Heinze et al., 2007). Furthermore, GlyRα4 transcripts have been detected in the white matter tract of the developing spinal cord from mice. The avian α4 mRNA was also found in dorsal root ganglia and genital ridge. Although the function of GlyRα4 at its sites of expression is still unknown, homomeric α4 receptors are able to form fully functional glycine-gated Cl^−^ channels *in vitro* that can be antagonized with strychnine (Harvey et al., 2000).

GlyRβ transcripts are widely expressed also in organs where no α subunit is present. The β expression starts around E14 but increases rapidly after birth (Malosio et al., 1991b). The β subunit is part of the adult GlyR complex, which is composed of 2α and 3β subunits (Grudzinska et al., 2005) with α1β representing the major form of heteromers. The function of the β subunits in tissues lacking GlyRα subunits is still enigmatic. Synaptic GlyRs are clustered by gephyrin via an interaction motif in the TM3-4 loop of the β subunits. Gephyrin is a scaffolding protein in the plasma membrane that mediates mobility of GlyRs and thereby contributes to the plasticity of inhibitory synapses (Fritschy et al., 2008; Calamai et al., 2009).

## Spontaneous mouse mutants of the *Glra1* and *Glrb* gene

**Figure 2:**
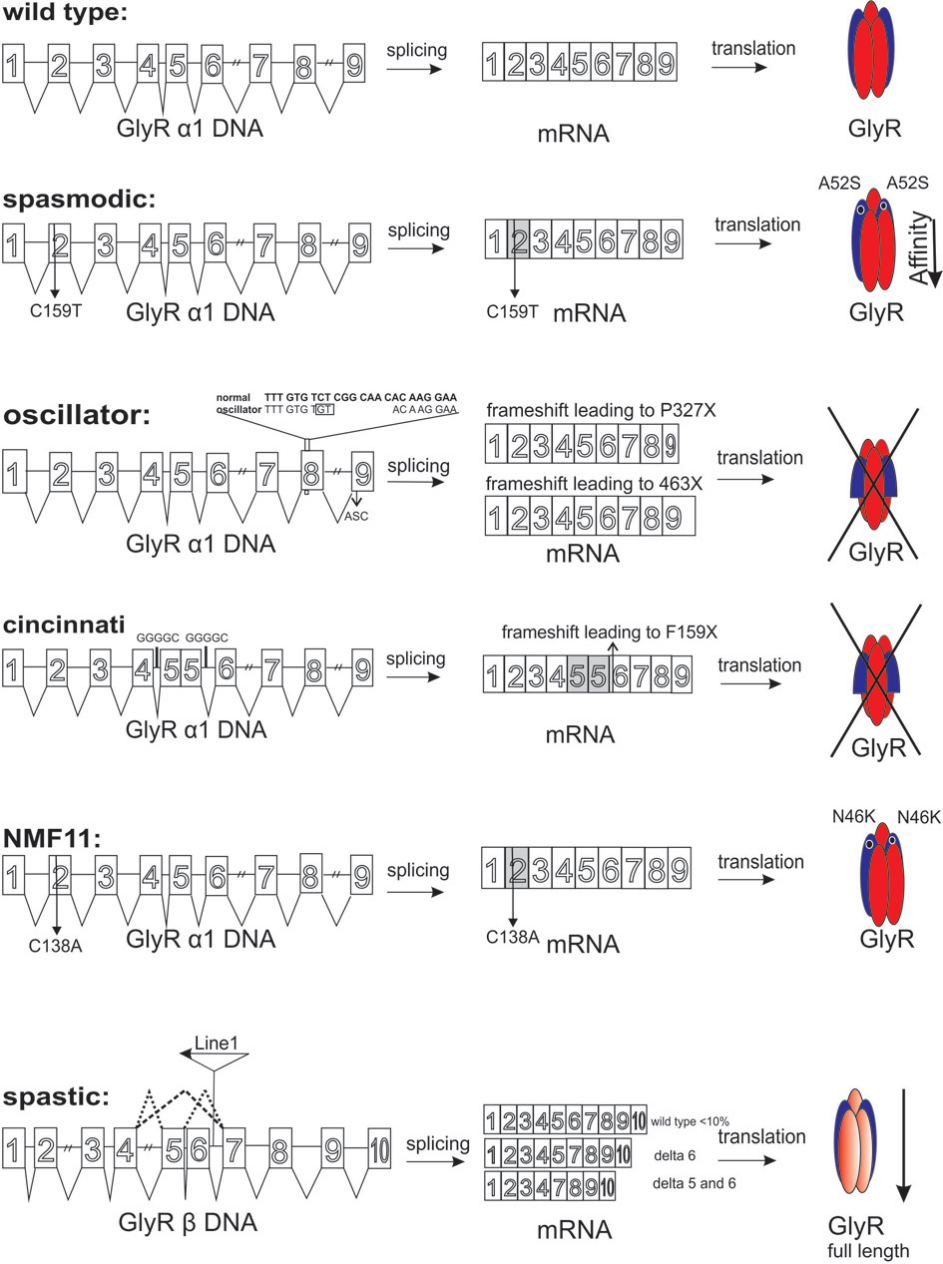
**Overview about GlyR mouse mutants from DNA level to the protein level**. The *Glra1* gene comprises 9 exons (*Glrb* 10 exons). Left column: SNPs are indicated by numbers. The C to T transition at position 159 in *spasmodic* mice is shown. The microdeletion in exon 8 of the *oscillator* mouse is listed by an insertion. In addition, exon 9 harbors an alternative splicing cassette (ASC) responsible for the generation of the two different mRNAs in the adult organism. For the *cininnati* mouse the intronic internal repeats (GGGGC) responsible for exon 5 duplication are shown. In the *Nmf* mutant a C to A transition in exon 2 of the *Glra1* gene was demonstrated. The insertion of the LINE1 element into intron 6 of the *Glrb* gene is marked. Aberrant splicing is indicated by dotted lines. Middle column: represents the different mRNAs, which are formed. SNPs are marked with numbers below the appropriate exon, e.g., *spd* C159T. Two mRNAs are generated in *oscillator* due to alternative use of the splice acceptor site in exon 9. Usage of this site leads to exclusion of the ASC (24 bp), non-usage generates an mRNA with the 24 bp ASC. *Cincinnati* harbors the exon 5 duplication with generation of a premature stop codon. The SNP C138A in the *Nmf11* mouse is marked. As a consequence of the LINE1 insertion into intron 6 of the *Glrb* gene, abberant splicing with exon 6 and exon 5/6 exclusion are observed. Right column refers to the GlyR proteins. In *spasmodic* mice a GlyR harboring the A52S mutation decreasing glycine affinity is marked. The premature STOP codons in *oscillator* and *cincinnati* result in no expression of GlyRα1. The single amino acid exchange N46K in the *Nmf* mutant is due to the C to A transition in exon 2 of the *Glra1* gene. Abberant splicing of *Glrb* in the *spastic* mouse model results in a highly reduced number of full-length GlyRβ.

To date five spontaneous and recessively inherited mutations in mice serve as models for human *hyperekplexia*. Four mutations occurred without any manipulation, such as *spasmodic* (*spd*), *oscillator* (*spd*^*ot*^), *cincinnati*, and *spastic* (*spa*) (Figure 2, Table 1). The fifth spontaneous mutation *nmf11* was induced by ENU (N-ethyl-N-nitrosourea), which is a powerful chemical mutagen that offers the possibility to induce point mutations genome-wide with a high frequency (Balling, 2001). This part will discuss the spontaneous mutations with regard to symptoms and progression of the disease, the genetic location of the mutations, and the resulting failure at the protein level being responsible for the phenotype of affected mice.

**Table 1:** **Spontaneous and induced GlyR mouse mutants**.

**Mouse mutant**	**Gene**	**Chromosome**	**DNA level**	**Protein level**	**Physiological effect**	**Lifespan**	**Further characteristics**	**References**
*Spasmodic* (spontaneous)	*Glra1*	11	G159T (exon 3)	A52S^*^	Decrease in glycine binding affinity	Normal symptoms start in third postnatal week	–	Ryan et al., 1994; Saul et al., 1994
*Oscillator* (spontaneous)	*Glra1*	11	Microdeletion of 9 bp and microinsertion of 2 bp (exon 8)	P327X^*^	Loss of function (NULL mutation), no synaptic localization	Lethal about three weeks symptoms start in second postnatal week	Rescue of GlyRα 1 function has been shown *in vitro*	Buckwalter et al., 1994; Kling et al., 1997; Villmann et al., 2009a
*Cincinnati* (spontaneous)	*Glra1*	11	Duplication of exon 5	F159X^*^	Loss of function (NULL mutation), no synaptic localization	Lethal about three weeks symptoms start in second postnatal week	–	Holland et al., 2006
*Nmf11* (induced)	*Glra1*	11	C138A (exon 3)	N46K^*^	no physiological data synaptic localization	Lethal about three weeks	ENU induced	Traka et al., 2006
*Spastic* (spontaneous)	*Glrb*	3	Insertion of LINE 1 element (intron 6)	less full-length GlyRβ	Deficient mRNA splicing with functional β mRNA levels being decreased to approximately 10%	Lethal about three weeks symptoms start in the second postnatal week	Backcross strain F1(C57BL/6J × C3H/HeJ) × C57BL/6J (C3H) show milder symptoms and mostly survive the critical age	Kingsmore et al., 1994; Mulhardt et al., 1994; Becker et al., 2012

* Amino acid positions refer to the mature protein.

### The *spasmodic* mouse mutant—a missense mutation

Homozygous *spd*/*spd* mice develop hyperekplexia like phenotypes characterized by an exaggerated acoustic startle reflex, high tremor, and an impaired righting reflex. Symptoms start in the third week after birth. Before this period, *spd*/*spd* mice are phenotypically normal and are indistinguishable from heterozygous or wild type siblings. No progression within the symptoms can be observed with age and homozygous mutants do have a normal life expectancy (Lane et al., 1987).

**Figure 3:**
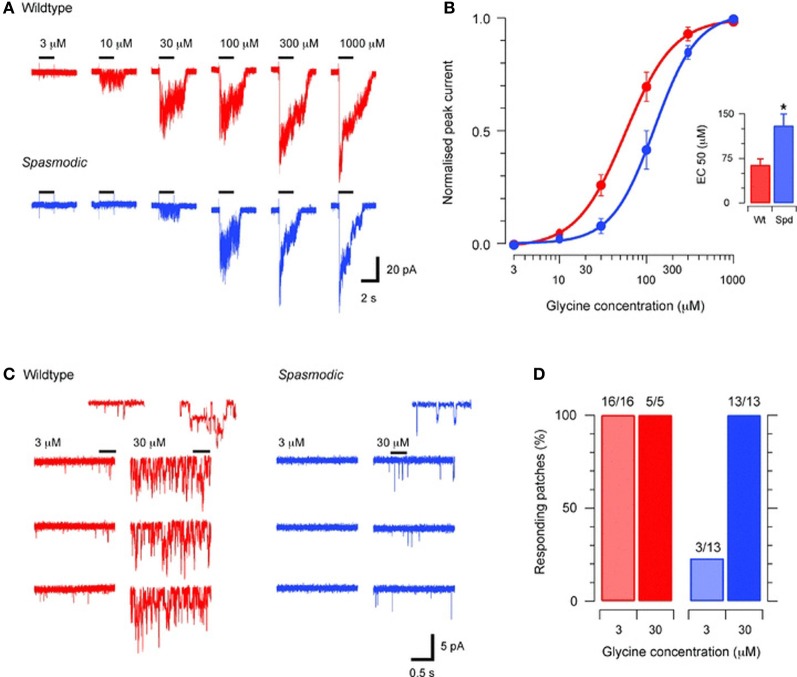
**Agonist sensitivity in *spasmodic* GlyRs recorded from superficial dorsal horn neurons [from Graham et al. (2011)]**. **(A)** Decreased glycine-evoked currents recorded in excised membrane patches from *spasmodic* SDH neurons compared to wild type using the same glycine concentration. **(B)** Concentration–response plots for wild type and spasmodic patches (*n* = 8 and 15, respectively) are shown. The concentration–response curve is shifted to rightwards for *spasmodic* patches. Inset summarizes EC_50_ values for spasmodic and wild type receptors. **(C)** Representative glycine responses for wild-type (left) and *spasmodic* (right) patches to bath application of low and high glycine concentrations. In wild type patches brief single channel openings are evoked by low glycine concentrations and high concentrations evoke multiple channel openings (inset shows channel openings on expanded time scale). In *spasmodic* patches no single channel events are observed at low glycine concentrations. At high glycine only a few openings are observed. **(D)** Plots summarizing results from experiments in **(C)**. Only 3/13 *spasmodic* patches responded at low glycine concentration, demonstrating again the lower affinity of this mutant to the agonist compared to wild type. ^*^P< 0.05.

A point mutation in the *Glra1* gene at chromosome 11, which leads to a change of an alanine into a serine at position 52 of the mature polypeptide sequence, has been found as the genetic reason for the phenotype in *spasmodic* mice (Ryan et al., 1994). The *spd* locus on chromosome 11 is linked by synteny homology to the human chromosomal region 5q21–q31, where the α1 subunit gene has been mapped (Shiang et al., 1993). A further but silent mutation was found at position 198 (Figure 2, Table 1) (Matzenbach et al., 1994; Saul et al., 1994). Although these animals develop a phenotype, the location of the protein seems not to be affected. The X-ray structure solved from homologous CLR protein AchBP defined various loop structures within the N-terminal domain of the protein that are involved in ligand binding (Brejc et al., 2001). The amino acid position A52S does not lie within this interface between two adjacent subunits, neither at the plus nor at the minus site of ligand binding. A52S only flanks the proposed assembly boxes and seems not to impair the overall protein content of GlyRα1 which is indistinguishable between wild type and homozygous *spasmodic* mice (Saul et al., 1994). In these channels the binding affinity of the agonists glycine, β-alanine and taurine is altered with a rightward shift in the glycine EC_50_ (Figure 3) (Saul et al., 1994; Graham et al., 2006, 2011). Excised patches from spasmodic superficial dorsal horn (SFDH) neurons show no single channel openings at low glycine concentration and only a few openings to high glycine concentrations compared to wild type neurons with multiple channel openings at the same glycine concentration. A detailed characterization of another point mutation at position 52 (A52C) within β1–β2 loop suggests that the microenvironment of A52 is responsible for different conformations of the β1–β2 loop and thereby hamper the agonist glycine from binding to the subunit interface (Pless and Lynch, 2009).

### The *oscillator* mouse—a frameshift mutation leading to complete loss of function

Homozygous *oscillator* mice represent a null mutation accompanied by a lethal phenotype. In contrast to *spasmodic* mice, *oscillator* mice always die within 3 weeks of age. Their neuromuscular symptoms like fine tremor and muscle spasm start at the age of 2 weeks and worsen progressively. The symptoms of this phenotype are highly comparable to strychnine poisoning in humans.

The genetic cause is also a mutation in the *Glra1* gene (Buckwalter et al., 1994). The *oscillator* allele *Glra1*^*spd−ot*^ exhibits a microdeletion of 9 bp plus a microinsertion of 2 bp resulting in a frameshift. Depending on differential use of two splice acceptor sites in exon 9 of *Glra1* (Figure 2, Table 1), two α1 polypeptide mutants are encoded, both of which lack TM4 (Malosio et al., 1991a). Inclusion of the alternative splice cassette resulted in a premature STOP codon within this 8 amino acid cassette thereby comprising a truncated α1 variant (*spd*^*ot*^-trc). The long splice variant, *spd*^*ot*^-elg, was generated from an exclusion of the alternative 24 bp and encodes 150 missense residues at the C-terminus (Villmann et al., 2009a).

**Figure 4:**
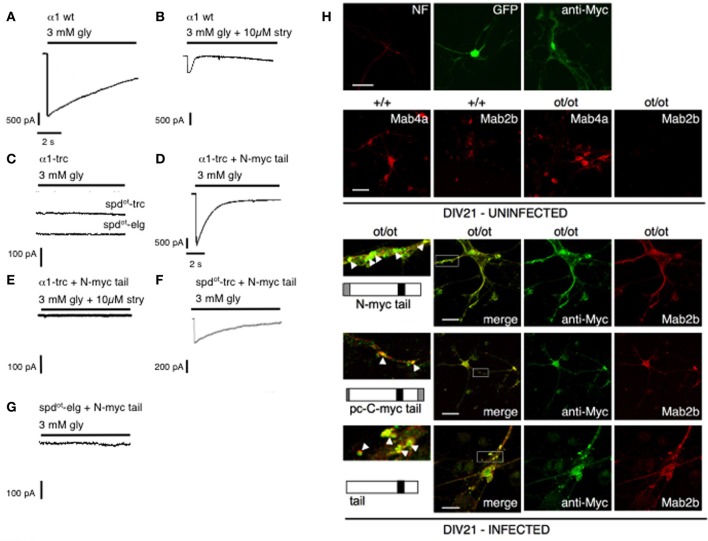
**GlyRs re-constituted from independent folding domains (modified from Villmann et al., 2009a)**. Rescue of function experiments of *spd*^*ot*^ constructs compared with truncated α1 variants. Using whole-cell recordings from transfected HEK293 cells, maximal glycine (gly)-gated currents were determined with 3 mM glycine: **(A)** α1A wt; **(B)** α1A wt blocked with strychnine (stry); **(C)** α1A-trc, *spd*^*ot*^-trc and *spd*^*ot*^-elg when expressed alone generated non-functional channels; **(D)** α1A-trc plus myc–α1–iD–TM4 restored functionality and was antagonized with strychnine **(E)**; **(F)** *spd*^*ot*^-trc plus myc–α1–iD–TM4 restored functional GlyRs but with less efficiency compared to α1-trc; **(G)** *spd*^*ot*^-elg plus myc–α1–iD–TM4 never generated functional channels. **(H)** Spinal cord neurons were infected with C-terminal domain constructs encoded on a pAAV vector. Comparison of infected (DIV21; bottom) and uninfected spinal cord neurons isolated from either homozygous wild type (+/+) or *oscillator* (*ot/ot*) mice (DIV21 uninfected). The α1-antibody Mab2b recognizes only the α1 variant, whereas pan-α recognizes all GlyRα variants. After infection with different constructs (myc–α1–iD–TM4, pc–α1–iD–TM4-myc, or α1–iD–TM4) infected neurons expressed endogenous GlyRα1 protein (bottom 3 panels, right pictures, depicted in red) that co-localized (merged pictures) with the appropriate C-terminal domains.

Due to the frameshift motifs important for receptor biogenesis and trafficking are destroyed (Sadtler et al., 2003). In the CNS of homozygous *oscillator* mutants, a complete loss of functional GlyRα1 protein neither *spd*^*ot*^-trc nor *spd*^*ot*^-elg was observed, characterizing *oscillator* as a functional null mutation of the *Glra1* gene (Kling et al., 1997). Using an independent domain approach *in vitro*, we were able show that the truncated GlyRα1 subunit variant *spd*^*ot*^-trc can efficiently be rescued by coexpression with an independent C-terminal tail domain representing the lacking GlyRα1 protein proportion (most of the TM3-4 loop, TM4, and the C-terminus). This functional complementation of GlyRs demonstrated the importance of the TM3-4 loop for biogenesis, clustering and pharmacological properties as demonstrated in transfected HEK293 cells and spinal cord neurons from *ot/ot* animals (Figure 4). Such reconstruction of a functional ligand-gated ion channel by independent folding domains resembles novel directions for compensatory mechanisms using gene therapeutical approaches (Villmann et al., 2009a). *In vivo* recordings in hypoglossal MNs of *homozygous oscillator* mice demonstrated a dramatic decrease in frequency and amplitude of mIPSCs together with a much slower decay time compared to heterozygous control mice. The fact of any glycinergic mIPSCs in the functionally null mutant *ot/ot* suggests a compensation via α2, α3, or α4 subunits expressed in MNs; however, these at distal dendritic locations expressed receptor clusters are not sufficient to sustain life (Graham et al., 2006).

### The *cincinnati* mouse—another frameshift mutation

*Cincinnati* is a spontaneous mutation in the *Glra1* gene with a similar phenotype to the other spontaneous mouse mutants already described. At 2 weeks of age, affected mice exhibit impaired righting reflex and tremor. This progress worsens till their death within 3 weeks of age as observed for *oscillator* mice (Figure 2, Table 1) (Holland et al., 2006). The mutant was recognized by the generation of multiple affected offsprings from one mating. Further breedings with obligate heterozygote animals with the strain C3H/HeCr1BR showed more mutants among the F1 generation. Further randomly crosses with the mentioned strain showed a recessively inherited autosomal inheritance.

Analysis of the genomic sequence did not show any insertions of LINE or SINE (long or short interspersed nuclear elements) elements as seen in *spastic*. But in introns 4 and 5 of *Glra1* gene a perfectly aligned repeat of the pentamer GGGGC was found. Very short direct repeats in adjacent introns are able to mediate exon duplication (Chen et al., 2005). The *cincinnati* mouse, indeed, shows two tandem copies of the 83 bp exon 5. This exon duplication in *Glra1* transcript results in a frameshift mutation with a novel generated premature stop codon (F159X) followed by a loss of GlyRα1 function comparable to the *oscillator* mutant. The predicted protein lacks the C-terminal 271 residues of the GlyRα1 protein. Thus, exon duplication is a common process contributing significantly to the evolution of functional complexity in the genome. Large deletions have also been observed in humans suffering from hyperekplexia with a deletion of exons 1–7 (Becker et al., 2006). Here, a complete loss of GlyRα1 is, however, much better tolerated by the human organism arguing for different compensation routes in humans compared to mice.

### The *spastic* mouse mutant—an aberrant splicing mutation

Homozygous *spa* mice manifest at the postnatal age of 2 weeks hyperekplexia-like severe neuromuscular symptoms, such as exaggerated startle response, rapid tremor, myoclonus, rigidity, and impaired righting reflex (Figure 2, Table 1) (Becker, 1990; Kingsmore et al., 1994; Mulhardt et al., 1994).

An insertion of a repetitive LINE1 element in intron 6 of the *Glrb* gene at chromosome 3 is the genetic reason for the neuromotor phenotype in *spa/spa* mice. This mutation leads to deficient mRNA splicing with functional β mRNA levels being decreased to approximately 10% (Kingsmore et al., 1994; Mulhardt et al., 1994). The mouse GlyR β subunit is encoded on chromosome 3q. The β subunit of the GlyR is known to be an important structural component of the pentameric GlyR complex for its synaptic location. Clustering of GlyRs at postsynaptic sites is enabled via an interaction of a binding motif in the intracellular TM3-4 loop of the β subunit to the scaffolding protein gephyrin. Moreover, recently it has been shown that GlyRβ is also involved in ligand binding, yet with low affinity (Grudzinska et al., 2005; Dutertre et al., 2012). It has been reported that within litters mild as well as severe phenotypes of homozygous *spa/spa* animals exist. A different level of mRNA expression has been ruled out (Molon et al., 2006). Following a differentiation of *spa/spa* animals into three groups, mice with early stage (M.e.s.), mice with recovery stage (M.r.s.), and severe phenotype (Sev), a compensatory effect with an up-regulation of the GlyRα1 mRNA has only been found for older recovered mice (M.r.s.) concomitantly with a higher GlyRα1 protein amount. In contrast, an up-regulation of the fetal glycine receptor GlyRα2 could never been observed. The higher amount of GlyRα1 protein can be one possible mechanism of compensation of GlyRβ loss accompanied by functional recovery observed in these mice, respectively. Furthermore, in mice with a severe phenotype apoptosis together with the loss of glycinergic interneurons in the ventral horn of the spinal cord have been found followed by a dramatic loss of MNs. Last could explain the paralysis and early death of these homozogous *spa/spa* population (Molon et al., 2006). Functional data from recordings in brainstem slices containing the hypoglossal nuclei further support this observation. Graham et al. have demonstrated that in *spa/spa* mice glycinergic inhibition in hypoglossal MNs is decreased without a dramatic change in receptor kinetics; however, whole-cell GlyR currents were picrotoxin sensitive. Picrotoxin-sensitivity did not result from homomeric GlyRα2 or GABAergic compensation as upregulation of GlyRα2 was not observed and all measurements have been performed in the presence of GABA_A_ receptor antagonist bicuculline. Thus, at least a distinct proportion of GlyRs in *spastic* mice results from homomeric GlyRα1 receptors. A synaptic location of homomeric α1 clusters was excluded since GlyR-mediated evoked IPSCSs from *spa/spa* and wild type animals showed the same picrotoxin sensitivity (Graham et al., 2006). Furthermore, a decrease in GlyR number at synaptic sites has been shown by different approaches, such as immunohistochemical or biochemical stainings (Becker et al., 1986; Callister et al., 1999). The described examinations so far only concentrated on the postsynaptic site of the glycinergic synapse. Muller et al. considered presynaptic effects in *spa/spa* mice. In the developing hypoglossal nucleus of *spastic* mice glycinergic presynaptic terminals decreased during the first two postnatal weeks whereas GABAergic terminals increased. In contrast, glycinergic terminals increase in inhibitory synapses in wild type mice during the same developmental period. Although this presynaptic adaptation of neurotransmitter content in response to the decrease of GlyRβ at the postsynaptic membrane demonstrates a crosstalk between pre- and postsynaptic elements in *spastic* mice, the observed GABAergic upregulation was not sufficient to compensate for defective glycinergic neurotransmission (Muller et al., 2008).

Interestingly, the reduced expression of GlyRβ at the protein level can be rescued by transgenic expression of an exogenous rat GlyRβ minigene. This minigene construct contained a rat GlyRβ cDNA, which was cloned into an expression vector suitable for driving brain-specific expression under the control of the rat neuron-specific enolase promoter (NSE). With this minigene a higher protein expression (approximately 25%) of full-length GlyRβ could be reached and a rescue of the symptoms has been observed (Hartenstein et al., 1996).

Homozygous mice (*spa/spa*) with a C57BL/6J (B6) background have strong symptoms and die at about 3 weeks after birth, while *spa/spa* animals of the backcross strain F1(C57BL/6J × C3H/HeJ) × C57BL/6J (C3H) show milder symptoms and mostly survive the critical age (von Wegerer et al., 2003). With ageing, these animals develop joint contractures and a vertebral arthopathy (Ziv et al., 1984), but their anatomical structures in spinal cord, CNS, or muscles are not altered or changed. The C3H/HeJ mouse strain seems to harbor an additional splicing factor or another modifying candidate gene, which enables *spa/spa* mice to sustain life. Very recently, a short nucleotide polymorphism (SNP) was localized in *Glrb* exon 6 at an exonic splicing enhancer (ESE) site, regulating exon skipping in *Glrb*. This ESE site is a binding site of the essential splicing regulatory protein SRSF1. Furthermore, the 5′ and 3′ UTR of the LINE1 were shown to be critical determinants for exon skipping, indicating that the LINE1 represents an efficient modifier of endogenous splicing phenotypes (Becker et al., 2012).

### The *Nmf11* mutation—an ENU induced point mutation

*Nmf11* homozygous mice show an *oscillator*-like phenotype with an early death within 3 weeks after birth. In contrast to *oscillator*, the *Nmf11* mutation was chemically induced by a powerful chemical mutagen N-ethyl-N-nitrosourea (ENU). ENU is used to induce point mutations genome-wide with a high frequency. The *Nmf11* mutation in mice courses a C-to-A transition of nucleotide 138, which results in the N46K substitution in the long extracellular N-terminal ligand-binding domain of the *Glra1* mature protein (Figure 2, Table 1) (Traka et al., 2006). The mutation does not affect *Glra1* mRNA and protein levels nor does it block the correct membrane targeting of the protein given that substantial amounts of the receptor were detected at synaptic sites of *Nmf11* homozygotes. The mechanism by which the *Nmf11* mutation impairs GlyR function is currently unknown. The mode of actions could either be due to differences in ligand binding or gating, respectively (Traka et al., 2006). Disturbances in receptor assembly seemed to be unfavorable, as well as a defect in oligomerization. The latter has been shown to be dependent on TM4 and aromatic residues within the other TMs (Haeger et al., 2010).

## Knock-out GlyR mouse mutants

A commonly used strategy to investigate the physiological role and significance of proteins is the generation of corresponding knock-out animals. Thereby the animal becomes deficient of the desired protein within either specific cell types or organs (conditional knock-out) or throughout the whole organism. Concerning the glycinergic system of inhibitory neurotransmission a variety of knock-out mice exist displaying deficiency of either the α2 or α3 subunit of the inhibitory GlyR. Naturally occuring mutations of the murine GlyRα1 subunit, as in the spontaneous mouse mutant *oscillator*, lead to loss of the respective protein, thus resembling the knock-out α1 situation (Kling et al., 1997). The absence of GlyRα1 exhibits the most severe phenotype since it is the subunit that is primarily responsible for glycinergic inhibition in the adult animals. It is widely expressed in brain stem and spinal cord, where the neuronal control of excitability is enabled by synaptic α1β heteromeric GlyR clusters. In contrast, deletions of either the GlyRα2 or α3 subunits resulted in mild phenotypes with no major morphological or molecular disturbances compared to *oscillator mice* (Harvey et al., 2004; Young-Pearse et al., 2006). Both knock-out mice will be discussed in more detail with regard to possible compensatory mechanisms.

### *Glra2* knock-out mice

The *Glra2* gene encodes the α2 subunit of the inhibitory GlyR. Two different variants α2A and α2B are known to be generated by alternative splicing (Kuhse et al., 1991). This subunit is capable of forming functional homopentameric receptors *in vivo*, which represent the embryonic and early postnatal form of glycinergic neurotransmission. The embryonic configuration is expressed throughout the nervous system and presumably located extrasynaptically (Flint et al., 1998). Moreover, due to the embryonic chloride ion distribution over the cell membrane, the α2 homopentameric receptors display an excitatory mode of action leading to a rise in intracellular calcium concentration (Flint et al., 1998). GlyRα2 homomeric receptors display a lower agonist affinity to glycine as compared to the α1 homopentamer. The potency of the agonists glycine, β-alanine and taurine has been described to be higher for the α2B variant (Miller et al., 2004). Mangin et al. investigated the kinetic properties of the α2 homopentameric GlyR at the single channel level. The homomeric α2 receptor type is slower by one or two orders of magnitude as compared to the α1/β heteropentameric receptor involved in synaptic glycinergic neurotransmission (Mangin et al., 2003). This was reflected by a slow onset, relaxation and desensitization and a low open probability upon synaptic-like application of glycine. These physiological characteristics render the α2 homomeric receptor inefficient for fast synaptic activation but comparatively suitable for activation via sustained and slow release of the agonist (Mangin et al., 2003).

Making use of *Glra2* knock-out mice the importance of this specific GlyR subunit for neuronal development during embryogenesis as well as for normal CNS function during adulthood has been investigated. First, there is no overt phenotype accompanied by the loss of the α2 subunit. The knock-out mice resemble normal weight compared to their wildtype littermates. Their life expectancy is unchanged and fertility is also not affected in the absence of the GlyRα2 subunit (Young-Pearse et al., 2006; Weiss et al., 2008). Young-Pearse et al. have investigated potential morphological alterations upon knock-out of GlyRα2. No striking abnormalities concerning the development and shape of spinal cord, cortex and retina have been found. Diameter and cell density of knock-out spinal cords were undistinguishable from the wild type situation. Calcium uptake studies of embryonic and neonatal cortical brain slices derived from knock-out animals confirmed the absence of functional GlyRs at that stage of development. While glycine had no effect on calcium uptake, stimulation by GABA induced a rise of intracellular calcium levels confirming unaltered GABAergic activity. The loss of glycinergic response was recovered at day P7 probably by expression of other GlyR subunits. However, despite the deficiency of electrophysiological responses to GlyR agonists such as glycine and taurine, the morphological development of cortices and cerebelli in knock-out animals seems to be unaltered (Young-Pearse et al., 2006).

In addition to its important role in brain stem and spinal cord, glycine also acts, besides GABA, as an inhibitory neurotransmitter in the neuronal networks of the retina (Pourcho, 1996) where it has been found in 40–50% of all amacrine cells (Marc, 1989). Within the variety of glycinergic amacrine cells, all α-subunits have been identified in specific synapses (Sassoe-Pognetto et al., 1994; Haverkamp et al., 2003, 2004; Heinze et al., 2007). Immunoreactivity of the GlyRα2 subunit was found to be evenly distributed across the inner plexiform layer representing the most frequent type of GlyRs in this area (Haverkamp et al., 2004).

### *Glra3* knock-out mice

The *Glra3* gene encodes the GlyRα3 subunit, which can be found within several regions of the CNS. Concerning its appearance in the spinal cord, it is evenly distributed throughout the superficial and deep laminae of the dorsal horn (Anderson et al., 2009). With the help of immunhistochemical experiments Harvey et al. could show that the GlyRα3 subunit can be co-stained predominantly in laminae II with gephyrin, which is necessary for synaptic localization. Moreover, it could also be co-stained with the α1 subunit of the GlyR, indicating that in these laminae II both subunit-specific glycinergic synapses exist, which contain either α1 or α3 and mixed synapses containing both α1 and α3. Lamina II is the brain region where most nociceptive afferent fibers terminate, which are involved in pain pathways (Harvey et al., 2004). Concerning these pathways, following prostaglandin E2 (PGE2) application a dramatic reduction in glycinergic inhibition of neurons within the dorsal horn of rat spinal cord, the first site of synaptic integration in the pain pathway was observed (Ahmadi et al., 2002). In turn, PGE2 levels increase in the CNS upon peripheral inflammation (Samad et al., 2001). First electrophysiological experiments with human embryonic kidney cells expressing the murine PGE2 receptor EP2 as well as the murine GlyRα3 or a mutant lacking a PKA consensus sequence revealed the putative mechanism of PGE2 activity via phosphorylation and thereby inhibition of GlyRα3. It has been demonstrated that GlyRα3 is selectively involved in spinal nociceptive processing. During inflammatory pain states, PGE2 disinhibits via this mechanism the spinal transmission of nociceptive input throughout the spinal cord dorsal horn to higher brain areas (Harvey et al., 2004). This signal cascade leads to a sensitization of primary afferents, thereby lowering thresholds for neuronal activation and increasing nociceptor responsivity. The observed phenotype results in increased responses to noxious stimulation termed hyperalgesia, and allodynia where normally non-noxious stimuli such as cooling, gentle touch, movement, and pressure are now perceived as being painful (Harvey et al., 2009). Understanding the role of GlyRα3 in pain processing pathways opened a new therapeutic window to use the α3 subunit as a target for treatment of chronic inflammatory pain. Ideally, therapeutic interventions could selectively increase the activity of GlyRs containing α3, which are down regulated via phosphorylation in inflammatory pain. Therefore and to get more insights into pain pathways an animal model was created: the *Glra3* knock-out mouse (Harvey et al., 2004).

Knock-out of the GlyRα3 subunit in mice, however, showed no obvious phenotype and was compatible with life. These mice normally gain regular body weight and display no histopathological abnormalities in brain and spinal cord. Moreover, no alterations in posture, activity, gait, motor coordination, tremor, and startle response have been observed (Harvey et al., 2004). Further studies have been carried out to characterize GlyRα3 knock-out mice concerning aspects of neurotransmission with regard to the sensation of different kinds of pain. Chronic pain can be broadly categorized into three types: inflammatory, neuropathic, and dysfunctional. Inflammatory pain arises from tissue damage and is associated with conditions such as arthritis. Neuropathic pain can arise from trauma in peripheral or CNS as a consequence of stroke or ischemia. The last type, dysfunctional pain, is a type of neural dysfunction like migraine characterized by a non-localized diffuse pain unaccompanied by either inflammation or nerve damage (Harvey et al., 2009). Experiments with α3 knock-out mice concerning the role of this GlyR subunit in different pain pathways also show that the inhibition of glycinergic neurotransmission by PGE2 does not contribute to neuropathic pain after peripheral nerve injury. This supports the idea that inflammatory and neuropathic pain involve different mechanisms of central sensitization (Hosl et al., 2006). To get more insights Harvey et al. investigated GlyRα3 and its potential role in other clinically relevant pain models as well as in neuropathic and visceral pain. GlyRα3^−/−^ showed no difference in the induction of thermal pain sensitization following CFA injection, but recovered much more quickly compared to the wild type. Yet, there is no clear role in other inflammatory (treatment with capsacin and carrageenan), neuropathic or visceral pain models. Thus, GlyRα3 may play an important role in mediating PGE2 induced sensitization, but only in certain pain states (Harvey et al., 2009). Experiments concerning development of GlyRα3 synapses contribute to previous findings. Any basal nociceptive hypersensitivity is absent in GlyRα3^−/−^mice, but GlyRα1 is still available for mediating synaptic inhibition at lamina II synapses but cannot be modulated by PGE2 signaling cascade (Rajalu et al., 2009).

## Knock-in mouse mutants

The approach of knock-in mice represents a powerful tool to look for physiological effects *in vivo* (Crestani et al., 2001). Knock-in mice have been generated to analyze the zinc modulation of inhibitory neurotransmission, which has been attributed to several residues in the N-terminus of GlyRα1 (Laube et al., 1995). Zn^2+^ homeostasis is thought to be important for brain development and function. Both, current responses of excitatory as well as inhibitory ligand-gated ion channels have been shown to be modulated by Zn^2+^ (Smart et al., 2004). Neutralization of aspartate 80 (D80) with a glycine or alanine eliminated Zn^2+^ potentiation of the glycine-gated responses (Lynch et al., 1998; Laube et al., 2000). Homozygous mice carrying the D80A mutation displayed a neuromotor phenotype with an increased muscle tone and massive tremor around P12, which persisted into the adulthood similar to that observed for the spontaneous mouse mutants *spasmodic* and *spastic*. In homozygous knock-in D80A mice (Hirzel et al., 2006) the potentiating effect of Zn^2+^ on glycine-gated currents recorded from cultured spinal cord neurons was reduced but not abolished using low concentrations. High concentrations of Zn^2+^ did not affect glycinergic inhibition. The residual Zn^2+^ potentiation observed in D80A knock-in mice may be due to other GlyRs than the α1 subunit. Immunocytochemical stainings showed massive labeling of extrasynaptic GlyRα2 arguing for residual Zn^2+^ potentiation via nonsynaptic GlyRs (Laube et al., 1995; Hirzel et al., 2006).

To study the pathomechanisms of human hyperekeplexia in mice, Becker et al. introduced a human dominant mutation tg271Q into the *Glra1* gene (Becker et al., 2002). Binding of the high affinity antagonist strychnine was unaffected, but glycine affinity was reduced in spinal cord of tg271Q. Similarly, *in vitro* studies on R271Q demonstrated decreased agonist affinity accompanied with diminished glycine-gated currents (Langosch et al., 1994; Rajendra et al., 1994). Whole-cell patch-clamp studies showed a decrease in glycinergic neurotransmission to 69%. Interestingly, amplitudes of electrically evoked IPSCs from spinal cord neurons were also reduced for GABA_A_-receptor-mediated currents, which was not due to a lower level of GABA_A_ receptor expression. Transgenic animals developed spontaneous tremor episodes from postnatal day 14 on with exaggerated startle responses to noise or touch. These data showed for the first time that a hyperekplexia-like phenotype is specific for the hyperekplexia associated mutated GlyR transgene. Besides the importance of GlyRs for motor coordination, these receptors are also targets for ethanol, longer chain alcohols and anaesthetics. Ethanol enhances glycine-gated responses and leads to loss of the righting reflex (LORR) in mice (Findlay et al., 2002). This effect can be antagonized with the glycine antagonist strychnine (Williams et al., 1995). Several *in vitro* studies led to the identification of distinct residues involved in ethanol potentiation (Mihic et al., 1997). S267N located in the ion channel pore is one of the key residues for ethanol modulation and has also been found in patients that suffer from hyperekplexia (Becker et al., 2008). This mutant displayed differences in agonist potency as well as ethanol modulation and demonstrated that a disease-associated *Glra1* mutation harbors the ability to alter drug responses. Similarly, the potentiating effect of ethanol was highly diminished in S267N knock-in mice. In contrast to the *oscillator* mutation, GlyRα1 protein expression in S267N knock-in animals was indistinguishable from wild-type levels. Glycine responses measured by uptake of glycine-stimulated chloride uptake from synaptoneurosomes from spinal cord and brainstem were decreased in heterozygous animals but comparable to heterozygous *oscillator* mice (Findlay et al., 2003). Muscimol-stimulated ^36^Cl^−^ uptake was unchanged arguing that GABA_A_ receptor activity stayed unaffected. Thus, GABA_A_ receptors are not attributable for a compensatory effect of the disturbed GlyR inhibition (Findlay et al., 2003). Behavioral tests, however, displayed an enhanced acoustic startle response (Findlay et al., 2005). Moreover, homozygous S267N knock-in mice resulted in lethality 3 weeks after birth similar to the functional null mutation (Kling et al., 1997; Findlay et al., 2003). This observation provided the first evidence that a point mutation does have a similar impact on the phenotype as the null allele. Homology analysis with mutated GlyRs on the GLIC structure searching for other amino acids, which are modulated by ethanol came up with two more candidates Q266I and M287L (Blednov et al., 2012; Borghese et al., 2012). Residue Q266 has also been found in a patient suffering from hyperekplexia (Milani et al., 1996). In knock-in mice both residues behave different to ethanol modulation. M287L reduces ethanol potentiation whereas Q266I abolished the ethanol potentiating effect. Physiological levels of Zn^2+^ have been shown to enhance ethanol potentiation of glycine-gated responses (McCracken et al., 2010). Although Zn^2+^ potentiation of glycine responses was unaffected, Zn^2+^ potentiation of the ethanol response was abolished in M287L. Glycine-induced maximal currents were reduced in isolated brain stem neurons similar to measurements in *Xenopus* oocytes. In addition, ethanol potentiation was absent in brainstem neurons isolated from homozygous knock-in mice. Radioligand binding assays demonstrated a reduced displacement of glycine by strychnine for knock-in animals, but flunitrazepam binding was not affected similar to previous findings in S267N knock-in mice (Borghese et al., 2012). These data again argue for lack of GABAergic compensation in GlyR-deficient mouse models. Behavioral characterization demonstrated an enhanced acoustic startle response for both mutants Q266I and M287L as observed for S267N, which is a hallmark for disturbances in glycinergic inhibition (Findlay et al., 2005; Blednov et al., 2012). A progressive phenotype starting from postnatal day 10 with massive muscle tremor and loss of motor control resulted in lethality between the 3 (Q266I) and 14 weeks (M287L) of life.

## Receptor assembly from independently folding domains

Receptor domains display high homology to other kinds of proteins, which exhibit different functions in their natural surrounding. The N-terminal part of CLRs for example shows high homology to the AchBP from the snail *Lymnea stagnalis*(Brejc et al., 2001). Similarly, the excitatory glutamate receptors represent a mosaic with homology of the ligand binding domains to bacterial periplasmic binding proteins (LAOBP, lysine-arginine-ornithine binding protein and LIVBP, leucine-isoleucine-valine binding proteins). LIVBP homology has been found in the far N-terminal domain of GluRs important for receptor assembly. In addition, the pore domain represents high homology to the ion channel domain of K^+^ channels (Villmann and Becker, 2007). Several studies on domain swapping have shown that homologous domains can be exchanged between different receptor subclasses without dramatic changes in ion channel properties (Strutz et al., 2002; Hoffmann et al., 2006; Villmann et al., 2008). Reconstitution of functionality from independent folding domains seemed to be a chance to gain-of-function for truncated receptor proteins. The mouse mutant *oscillator* was used as a model system since the underlying mutation, a microdeletion, results in a premature STOP codon. This mouse mutant represents a functional null allele (Kling et al., 1997). *In vitro* experiments demonstrated that the coexpression of truncated receptor protein together with an independent “tail” construct representing the lacking proportion of the GlyRα1 protein, most of the TM3-4 loop, TM4, and the C-terminus rescued the GlyR function to about 10–50% of wild type activity. An infection of spinal cord neurons isolated from homozygous oscillator mice with an adeno-associated viral vector encoding the independent tail construct induced an expression of the α1 antigen compared to uninfected cells lacking α1 expression (Figure 4) (Villmann et al., 2009a).

This view on GlyR assembly from different folding domains toward a functional receptor will be a useful tool for restoration of ion channel function in other kinds of diseases such as the idiopathic generalized epilepsies, where mutations in genes encoding the postsynaptic inhibitory GABA_A_ receptor subunits resulting in protein truncation have been identified.

## Possible routes of compensation

Mutations in GlyR genes *Glra1* and *Glrb* result in a neuromotor phenotype similar to patients suffering from hyperekplexia. That is why mice serve as excellent models for this human neuromotor disorder. The variety of mutations in mice comprises point mutations, microdeletions, duplications, and insertions. Human patients usually get treated with clonazepam, which helps via an enhancement of GABAergic responses to compensate for the lack or decrease of glycinergic inhibition. Although GABAergic compensation is also favored in mice, different experiments failed to show GABAergic upregulation at the level of expression. No changes in radioligand binding studies using brainstem or spinal cord membrane preparation from tissue have been observed meaning that the number of GABA_A_ receptors expressed in these membranes is unchanged (Becker et al., 2002; Blednov et al., 2012). Pharmacological studies on knock-in mice demonstrated that GABA_A_ receptor modulators such as flurazepam and pentobarbital induced an increase in LORR in addition to an increase in GlyR sensitivity to pentobarbital. Also picrotoxin-induced convulsions differed in knock-in mice suggesting changes in GABA_A_ receptor function (Blednov et al., 2012). An enhancement of GABAergic maximal current amplitudes has been shown in *spastic* mice, but in knock-in mice no functional differences were observed for GABA_A_ receptors (White and Heller, 1982; Findlay et al., 2003). Other compensatory effects via nAChR or NMDA receptors analyzed by ketamine or ethanol action used in behavioral studies of GlyR knock-in mice are difficult to interpret as these modulators at various concentrations act also directly on these classes of ligand-gated ion channels. What we have learned from knock-in mice is that there is very limited or no compensation. Moreover, *in vitro* studies on a human mutation with moderate effects on GlyR functionality could result in a severe impairment of glycinergic neurotransmission associated by lethality *in vivo*.

For the spontaneous mutations affecting one of the GlyR subunits present in the adult receptor complex, α1 or β, compensatory mechanisms have been described in detailed physiological recordings. The rise and decay times of mIPSCs were similar between *spa/spa* and controls. Therefore, changes in GlyR distribution seem unlikely, but the lower frequency is in line with less α1β clusters due to lower transcription rate of full-length β subunits. Compensation in *spa/spa* via homomeric α1 was favored as one possible mechanism; however, such homomeric α1 receptors have never been observed at synaptic sites (Graham et al., 2006). As a consequence of low GlyRβ numbers at the postsynaptic side in *spastic* mice, glycinergic presynaptic terminals decrease demonstrating an adaption process the second postnatal week (Muller et al., 2008). Recently, Becker et al. have demonstrated that mouse line background could also have a compensatory effect on the appearance of the *spastic* phenotype. In addition to the insertion of a LINE1 element in intron 6 of the *Glrb* gene resulting in changes in endogenous splicing, a SNP in exon 6 functions as an ESE important for binding of the splice factor SRSF1. Minigene experiments showed that mis-splicing of the β-subunit could be rescued by the A/G transition in exon 6 present in the C3H/HeJ mouse background but absent in C57BL/6J where the *spastic* mutation was originally described (Becker et al., 2012). In *ot/ot* animals slow mIPSCs with very low amplitudes suggest some remaining GlyR clusters present at distal dendritic locations. Although the expression of α2, α3, and α4 in *oscillator* is normal, the compensatory GlyR clusters are not sufficient to sustain life (Graham et al., 2006). In summary, some sort of compensation seem to exist in various mouse models for hyperekplexia, but neither upregulation of GABAergic responses nor compensation by other GlyR subunits are able to rescue glycinergic dysfunction in mouse spinal cord. Thus, disturbances in glycinergic inhibition perturb a neuronal network with the GlyR playing a key role in this network.

## Conclusions

Functional inhibitory GlyRs are important for neuromotor behavior. Disturbances in the glycinergic synaptic circuit lead to severe startle reactions in response to tactile or acoustic stimuli. Spontaneous mutations and knock-in mice carrying human hyperekplexia mutations serve as models to understand the human neuromotor phenotype. But why do some mutations in the GlyR genes (*Glra1* or *Glrb*) in rodents result in a mild and others in a lethal phenotype? Are there differences in compensatory mechanisms? The strength of a phenotype could result from mouse line background as recently shown for the two hit splice mutation in the mouse mutant *spastic*. Furthermore, the functional effect depends on the location of the mutation. Here, the increasing knowledge on the molecular structure of CLRs during the last decade opened novel windows for an interpretation of functional data with the help of homologous models. Interestingly, knock-out mice of the GlyRα2 or α3 demonstrated normal life span. In contrast, the functional null allele of the *Glra1* gene resulted in lethality at the age of 3 weeks when the developmental shift toward the adult receptor complex α1β is completed. Therefore, there is no doubt that heteromeric α1β GlyRs play the most important role for fast inhibitory neurotransmission processes in motor function. So far premature truncation of GlyRα1 could only be compensated when the lacking protein proportion was coexpressed at least *in vitro*. An assembly of functional GlyRs from independent domains seems to overcome processes like nonsense-mediated decay or when translated ER-mediated degradation. But the existence of similar coordinated assembly processes *in vivo* has to be proven. According to the human mutations, all described GlyRα1 knock-in studies exhibited similar functional differences on GlyRs compared to *in vitro* data obtained from either transfected cell lines or oocyte recordings. Thus, mice seem to be an excellent model system to study hyperekplexia. Mice carrying human mutations showed an onset of symptoms as massive tremor and startle attacks with the age of 15 days when developmental changes in GlyR subunit composition are almost completed. Treatment of human patients with clonazepam favored an up-regulation of the GABAergic neurotransmission. Therefore, most studies looking for compensatory mechanisms in mice concentrated on the receptor configuration at the postsynaptic side. All physiological studies so far have, however, strikingly shown that the central role of the adult isoform α1β cannot be compensated by another GlyR receptor configuration neither by α3β, homomeric α1, α2 nor distinct GABA_A_ receptors. Although there is still requirement to search for subunit-specific blockers of either α1, or α2, or α3, and β, to improve the interpretation of physiological data, our knowledge on the differences in the kinetics of GlyR subpopulations has amazingly increased during the last years. Yet, the complexity of synaptic circuits *in vivo* as well as synaptic adaption processes are far away from being understood. One future direction of research is trying to identify so far unknown regulatory elements or proteins responsible for synaptic adaptation at the inhibitory synapses during glycinergic disturbances. It has been shown that presynaptic terminals and postsynaptic elements communicate with, e.g., an adaptation of less presynaptic glycinergic terminals in response to less GlyRβ at the postsynapse in the mouse mutant *spastic*. GABAergic terminals instead increase but GABA_A_ receptor numbers stay unaffected. The various mouse models discussed here demonstrate that the current hypothesis of GABAergic compensation for a failure in glycinergic neurotransmission has to be carefully scrutinized. An enhancement of the GABAergic responses by treatment with clonazepam is therefore unlikely to explain the massive improvement of symptoms observed in human patients. Thus, the described mouse models in combination with modern imaging techniques and proteomics serve as excellent tools to close the gap between adaptation and remodeling of inhibitory synapses *in vivo* under physiological and pathological conditions.

### Conflict of interest statement

The authors declare that the research was conducted in the absence of any commercial or financial relationships that could be construed as a potential conflict of interest.
